# Mechanical strength of a new plate compared to six previously tested opening wedge high tibial osteotomy implants

**DOI:** 10.1186/s40634-019-0209-1

**Published:** 2019-11-07

**Authors:** Arnaud Diffo Kaze, Stefan Maas, James Belsey, Alexander Hoffmann, Romain Seil, Ronald van Heerwaarden, Dietrich Pape

**Affiliations:** 1 Faculty of Science, Technology and Communication, University of Luxembourg, 6, rue R. Coudenhove-Kalergi, L-1359 Luxembourg, Luxembourg; 2 Department of Orthopedic Surgery, Centre Hospitalier de Luxembourg, L-1460 Luxembourg, Luxembourg; 3 Cartilage Net of the Greater Region, 66421 Homburg/Saar, Germany; 4 Kliniek ViaSana, Centre for Deformity Correction and Joint Preserving Surgery, Mill, 1, 5451 AA Hoogveldseweg, Netherlands; 5 Sports Medicine Research Laboratory, Public Research Centre for Health, Centre Médical de la Fondation Norbert Metz, 76 rue d’Eich, L-1460 Luxembourg, Luxembourg; 6 Department of Sport, Exercise & Health, University of Winchester, Sparkford Road, Winchester, S022 4NR UK

**Keywords:** High tibial osteotomy (HTO), Osteoarthritis, FlexitSystem, Activmotion, TomoFix, PEEKPower, ContourLock, iBalance, Permanent deformation, Correction angle, Biomechanics, Mechanical stiffness, Static strength, Fatigue strength

## Abstract

**Background:**

This study aimed to assess the mechanical static and fatigue strength provided by the FlexitSystem plate in medial opening wedge high tibial osteotomies (MOWHTO), and to compare it to six previously tested implants: the TomoFix small stature, the TomoFix standard, the ContourLock, the iBalance, the second generation PEEKPower and the size 2 Activmotion. Thus, this will provide surgeons with data that will help in the choice of the most appropriate implant for MOWHTO.

**Methods:**

Six fourth-generation tibial bone composites underwent a MOWHTO and each was fixed using six FlexitSystem plates, according to standard techniques. The same testing procedure that has already been previously defined, used and published, was used to investigate the static and dynamic strength of the prepared bone-implant constructs. The test consisted of static loading and cyclical loading for fatigue testing.

**Results:**

During static testing, the group constituted by the FlexitSystem showed a fracture load higher than the physiological loading of slow walking (3.7 kN > 2.4 kN). Although this fracture load was relatively small compared to the average values for the other Implants from our previous studies, except for the TomoFix small stature and the Contour Lock. During fatigue testing, FlexitSystem group showed the smallest stiffness and higher lifespan than the TomoFix and the PEEKPower groups.

**Conclusions:**

The FlexitSystem plate showed sufficient strength for static loading, and average fatigue strength compared to the previously tested implants. Full body dynamic loading of the tibia after MOWHTO with the investigated implants should be avoided for at least 3 weeks. Implants with a wider T-shaped proximal end, positioned onto the antero-medial side of the tibia head, or inserted in the osteotomy opening in a closed-wedge construction, provided higher mechanical strength than implants with small a T-shaped proximal end, centred onto the medial side of the tibia head.

## Background

Medial open-wedge high tibial osteotomy (MOWHTO) is a common intervention used for the treatment of medial compartment gonarthrosis with varus malalignment in young and active patients (Amendola and Bonasia 2010; Pape et al. 2004). The maintenance of primary stability after MOWHTO depends on factors associated with the surgical technique and the implants used (Brinkman et al. 2008; Lobenhoffer and Agneskirchner 2003; Spahn et al. 2006; Spahn et al. 2007). Precise preoperative planning and high primary fixation stability of the implant are required for a good outcome (Pape et al. 2004). Numerous implants for MOWHTO are available on the market, which have different shapes and varying biomechanical and material properties. It is important to quantify and compare the stabilising effect of these implants to assist surgeons in the choosing of the most appropriate implant from a mechanical point of view. Diffo Kaze et al. performed biomechanical studies (Maas et al. 2013; Diffo Kaze et al. 2015; Diffo Kaze 2016; Diffo Kaze et al. 2017) that compared the following six implants: the TomoFix small stature (TomoFix sm) and TomoFix standard (TomoFix std) plates of Synthes Gmbh (Oberdorf, Switzerland), and the ContourLock plate, the iBalance implant and the second generation PEEKPower plate of Arthrex (Munich, Germany), and the size 2 Activmotion plate of Newclip Technics (Haute*-*Goulaine, France) (Tables 1 and 2, groups I to VI). The FlexitSystem of Neosteo (Nantes, France) is a new HTO implant (Table 2, Group VII) that is pre-contoured to fit the medial proximal tibia, similar to the other five previously tested plates. The iBalance implant is inserted centrally into the osteotomy gap on the medial side of the tibia head. Except for the size 2 Activmotion, which is positioned onto the antero-medial surface of the tibia head, all the previously tested implants and the FlexitSystem have their proximal part centred onto the medial surface of the tibia head. The proximal part of the FlexitSystem is dimensionally comparable to the other T-shaped implants, but has only three proximal screws whereas the others have four.

**Table 1:** Different HTO implants considered in the study (Groups I, II and III)

Groups	Implant picture	Material	Design/fixation principle
TomoFix std (Group I)		Titanium	Long T-shaped internal fixator with uniaxial angle stable locking screws. The five proximal locking screws are bicortical and the three distal are monocortical
PEEKPower (Group II)		Carbon-fiber reinforced polyetheretherketone (PEEK)	T-shaped internal fixator, shorter than the TomoFix plate, with angle stable multidirectional screws. The proximal screws are monocortical and fixed in the cancellous bone and the distal screws are bicortical.
iBalance (Group III)		Non-absorbable PEEK	Spacer inserted in the osteotomy wedge attached to the tibia by PEEK screws. The distal screws are fixed in the cancellous bone and the distal in the cancellous bone until the cortical opposite bone

The implants have different shapes. All the implants are centred onto the medial surface of the tibia head. The iBalance implant is inserted centrally into the medial side of the tibia head

**Table 2:** Different HTO implants considered in the study (Groups IV to VII)

Groups	Implant picture	Material	Design/fixation principle
TomoFix sm (Group IV)		Titanium	Same fixation principle with the TomoFix std. Geometry adapted from the TomoFix std. to patient with small stature
ContourLock (Group V)		Titanium	Short spacer plate with large proximal part and angle stable multidirectional screws. The proximal screws are monocortical and fixed in the cancellous bone and the distal screws are bicortical.
Activmotion (Group VI)		Titanium alloy	Internal fixator with eight monoaxial locking screws. The diaphyseal screws are bicortical while the epiphyseal are monocortical
FlexitSystem (Group VII)		Titanium alloy	T-shaped internal fixator, shorter than the TomoFix plates, with angle stable unidirectional screws. The proximal screws are monocortical and fixed in the cancellous bone and the distal screws are bicortical.

The implants have different shapes. All the implants are centred onto the medial surface of the tibia head, except for the size 2 Activmotion that is positioned onto the antero-medial side of the tibia head

In the present study, we aimed to compare the mechanical static and fatigue strength provided by the FlexitSystem against the six previously tested implants designed for the treatment of medial knee joint osteoarthritis, using a testing procedure that has already been defined, used and published (Maas et al. 2013; Diffo Kaze et al. 2015; Diffo Kaze 2016; Diffo Kaze et al. 2017). We hypothesized the following: (1) The bone-implant constructs with the FlexitSystem should fail due to the collapse of the opposite cortex. (2) Due to its similar positioning and T-shaped form as the TomoFix and the PEEKPower plates, but with only three proximal screws, the FlexitSytem should provide comparable or lower mechanical strength than the TomoFix and the PEEKPower plates. (3) This means that the FlexitSystem should be inferior to the iBalance, the Activmotion and the Contour Lock, based on the observations of the previous studies regarding the mechanical stability provided by implants (Maas et al. 2013; Diffo Kaze et al. 2015; Diffo Kaze 2016; Diffo Kaze et al. 2017).

## Methods

MOWHTO with FlexitSystem plates were performed on six large-size fourth generation composite analogue tibia bone models (Sawbones, Pacific Research Laboratories, Inc., Vashon, WA) in the same way by an experienced surgeon, according to standard techniques of the plate. The same standardised procedure, as used in the previously performed osteotomy tests (Maas et al. 2013; Diffo Kaze et al. 2015; Diffo Kaze 2016; Diffo Kaze et al. 2017), was used to prepare the specimens. The same loading protocols for static and dynamic tests, as for our previous studies, were employed to test the FlexitSystem specimens. Hence, the FlexitSystem (Table 2, Group VII) was able to be compared to the six previously tested implants (Tables 1 and 2; Group I to VI). Overall, the thirty-three specimens, whose test results were used in the present study, were subdivided according to the type of test performed, as indicated in Table 3. It has been shown that the loading of the specimens that was used for the experimental setup, corresponded to a realistic loading of the lower limb during the loading response phase of slow walking (Diffo Kaze et al. 2018).

**Table 3:** Specimen grouping and assignment, depending on used implants and the performed test

Performed test	Group I; *n* = 5 Specimens	Group II; *n* = 5 Specimens	Group III; *n* = 6 Specimens	Group IV; n = 5 Specimens	Group V; *n* = 5 Specimens	Group VI; *n* = 6 Specimens	Group VII; *n* = 6 Specimens
Static: single loading to failure test	TomoFix 1	PEEKPower 1	iBalance 1	TomoFix sm 1	Contour Lock 1	Activmotion 1	FlexitSystem 1
	TomoFix 2	PEEKPower 2	iBalance 2	TomoFix sm 2	Contour Lock 2	Activmotion 2	FlexitSystem 2
Dynamic: cyclic fatigue to failure test	TomoFix 3	PEEKPower 3	iBalance 3	TomoFix sm 3	Contour Lock 3	Activmotion 3	FlexitSystem 3
	TomoFix 4	PEEKPower 4	iBalance 4	TomoFix sm 4	Contour Lock 4	Activmotion 4	FlexitSystem 4
	TomoFix 5	PEEKPower 5	iBalance 5	TomoFix sm 5	Contour Lock 5	Activmotion 5	FlexitSystem 5
			iBalance 6			Activmotion 6	FlexitSystem 6

For the static tests, the specimens were subjected to a quasi-static compression,displacement-controlled single loading to failure at a speed of 0.1 mm/s. The dynamic tests consisted of load-controlled cyclical fatigue testing, with stepwise compression and sinusoidal (frequency = 5 Hz) loading, where the force amplitude of each step was kept constant by the feedback control of the force signal within the hydraulic machine. The lower compressive force limit of each load step was kept constant at 160 N. Starting at 800 N for the first step, the upper compressive force limit was increased stepwise by 160 N after *N* = 20,000 cycles if no failure occurred.

**Fig. 1:**
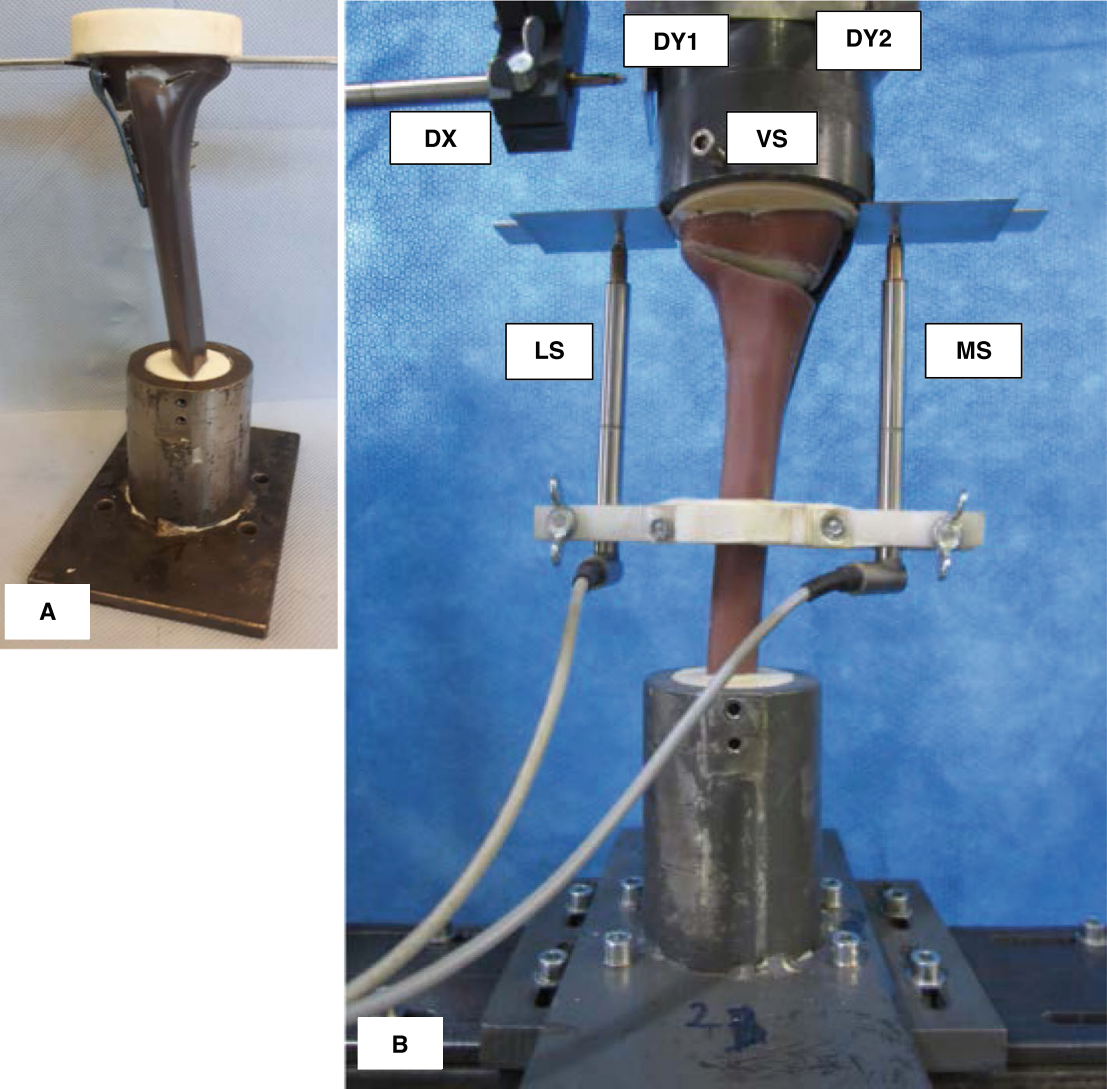
Specimen and sensors’ locations: **a** Specimen before mounting to hydraulic press. **b** Specimen under test. The lateral and the medial sensor (LS and MS) register the relative lateral and medial vertical displacements from the tibial head, while VS measured its vertical displacement. The sensors DX, DY1 and DY2 register the horizontal displacements of the tibial head; along the transverse axis for the first and the sagittal axis for the latter

Vertical loading was applied to the tibia head of the FlexitSystem specimens (Fig. 1-A), as with the previously tested implants, through a freely movable support, allowing for any horizontal motion in the transversal plane, using three freely rolling metal balls. The displacement in the frontal plane on the medial side of the tibia head was measured by a medial sensor MS. A second sensor LS on the lateral side measured the lateral displacement. Three displacement sensors DX, DY1, and DY2 were attached on the freely sliding support in order to measure the horizontal displacements of the tibia head in two perpendicular directions. A fifth displacement sensor VS embedded in the INSTRON machine measured the vertical displacement of piston (Fig. 1-B).

### Failure criteria

The following failure criteria (Table 4) that have already been used by Pape et al**.** (Pape et al. 2010) and considered in the previous studies were considered in the present study. The failure type 3 allow for a quantifying of the wobble degree, or stability, of the sample during the cyclic testing (Maas et al. 2013; Diffo Kaze et al. 2015; Diffo Kaze 2016; Diffo Kaze et al. 2017).

**Table 4:** Used failure types and their defining criteria (Maas et al. 2013; Diffo Kaze et al. 2015; Diffo Kaze 2016; Diffo Kaze et al. 2017)

Failure type	Criteria
1	Medial or lateral displacements of the tibial head in relation to the tibial shaft of more than 2 mm, equivalent to a rotation of more than 1.4 °. A counter-clockwise rotation corresponds to a valgus malrotation of the tibia head. This criterion can only be checked in the unloaded condition.
2	Visible collapse of lateral cortex. Small hairline cracks are not considered as failure.
3	Maximal displacement range of more than 0.5 mm within one hysteresis loop in the case of cyclic testing only.
4	Cracks of the screws of more than 1 mm

### Permanent deformation and deflection due to plastic deformation during the cyclic testing

**Fig. 2:**
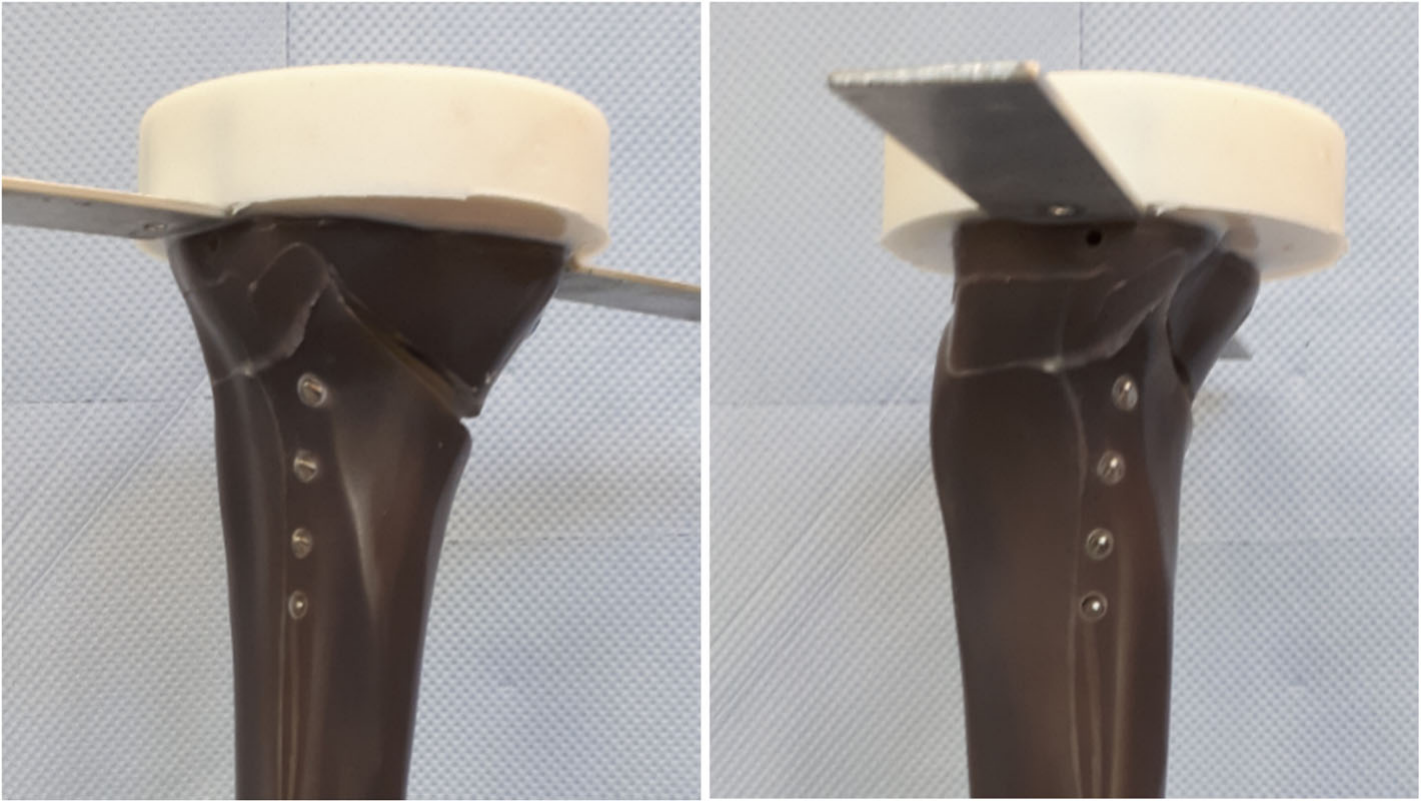
Fracture of the lateral cortex during static testing (FlexitSystem 1). The opposite cortex appeared to be the weak point of the bone-implant constructs

The permanent deformation, after unloading the specimen, results from plastic deformation. It was estimated as the irrecoverable displacement from the start of the tests at the minimal force of 160 N that was considered as nearly zero force. The permanent deflection angle *α*_*p*_ after the collapse of the contralateral cortex during the cyclic tests was determined using the method indicated by Diffo Kaze et al. (Diffo Kaze et al. 2015; Diffo Kaze 2016; Diffo Kaze et al. 2017). The deflection angle corresponds to a rotation of the tibia head relative to the shaft, which occurs in the frontal plane (Fig. 2). It was calculated as the deflection resulting from the absolute difference between the lateral and medial displacements. Permanent deflection angle *α*_*p*_, greater than 0.024 rad or 1.4°, corresponds to the occurrence of failure type 1 (Table 4).

### Stiffness of the specimens

The dynamic stiffness of the specimens was determined as the damage indicator during the cyclic tests. It was calculated as the ratio of peak to peak force ∆F to the measured peak to peak displacement ∆X in the same period

$$ \mathrm{K}=\frac{\Delta  \mathrm{F}}{\Delta  \mathrm{X}}. $$

The static stiffness, at the critical state when the damage of the specimen occurs, was calculated as the ratio of the corresponding damage load (F_Damage_) to the corresponding displacement (X_Damage_)

$$ \mathrm{K}=\frac{{\mathrm{F}}_{\mathrm{Damage}}}{{\mathrm{X}}_{\mathrm{Damage}}}. $$

### Statistical analysis

The number of specimens was limited due to financial reasons. There were neither final loads, displacements of the tibia head, nor number of cycles prior to failure that were predefined as reference quantities in the present study. Hence, no statistical analyses were performed within each group. The t-test for two independent samples was used to compare the ultimate loads, the displacements of the tibia head, the valgus malrotation, the lateral stiffness and the number of cycles prior to failure between the FlexitSystem group and the others. Statistical analysis was performed using Microsoft Excel 2010 software (Microsoft Corporation, Redmond, Washington, USA). All statistical tests were performed two sided. Statistical significance was considered at *p* <  0.05.

## Results

The same materials and methods from our previously performed and published studies were used for the specimens with the FlexitSystem plate (Group VII, Table 2) in the present study. Hence, the results obtained from all these studies were comparable. The published results of our previous studies (Groups I to VI, Table 2) are also presented here for comparison purposes.

### Static loading to failure

During the static testing, the FlexitSystem specimens failed by fracture of the contralateral cortex (Fig. 2), similar to all the previously tested specimens. The fractures were abrupt and no crack formation prior to the ultimate rupture was observed. No defects of the plates or screws were observed.

**Fig. 3:**
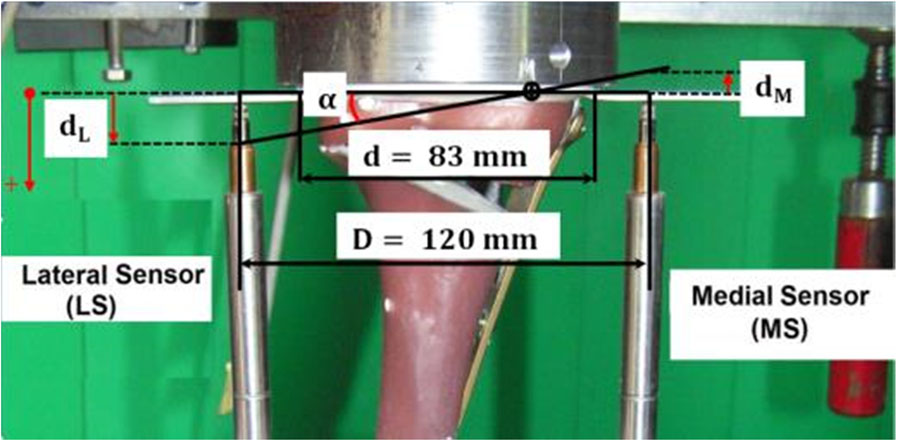
Valgus-malrotation of the tibia head. The lateral displacement d_L_ was assumed positive and the medial displacement d_M_ was assumed negative. The angle α represented the valgus-malrotation of the tibia head and was calculated by mean of the difference |***d***_***L***_ ***− d***_***M***_|

**Fig. 4:**
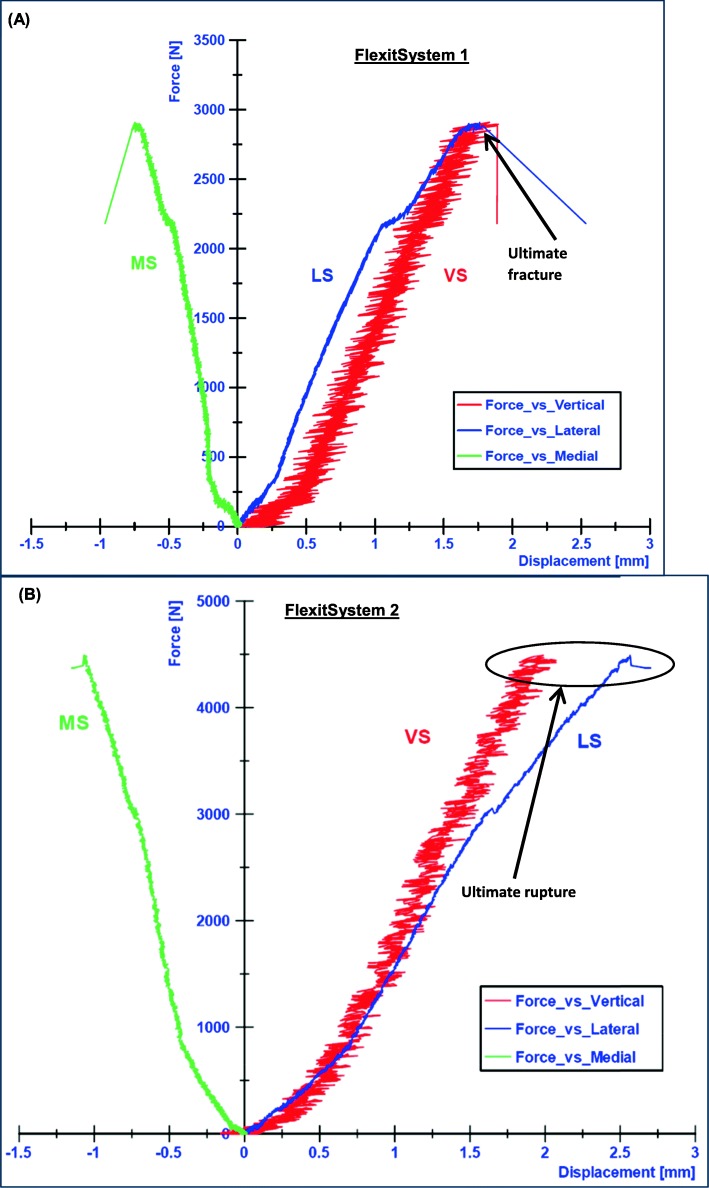
Static test results: **a** FlexitSystem 1, **b** FlexitSystem 2. The ultimate loads were considered as the approximate loads at the moment of collapse of the contralateral cortex respectively

The lateral side of the tibia head performed a downward movement, which was considered as positive, while the medial side performed an upward movement, which was considered negative. Thus, the medial displacements (MS) were negative and the lateral displacements were positive (Fig. 3). Consequently, a rotation of the tibia plateau in the frontal plane, valgus-malrotation, was observed. The lateral displacements had larger absolute amplitude than the medial displacements (Fig. 4).

For the FlexitSystem 1, the ultimate load was approximately 2.9 kN, which corresponded to medial and lateral displacements of about 1 mm and 2.5 mm respectively. For the FlexitSystem 2 the ultimate load was approximately 4.5 kN, which corresponded to an ultimate medial displacement of 1.15 mm and a lateral displacement of 2.7 mm (Fig. 4).

The results of the static tests performed on the FlexitSystem were summarised together with the results of our previous studies (Table 5) and have been reported here for comparison purpose.

**Table 5:** Static tests summary

Specimen	Crack / Ultimate load [kN]	Medial displ. at crack/ ultimate load [mm]	Lateral displ. at crack/ ultimate load [mm]	valgus-malrotation of the tibial head at crack/ ultimate load (°)	Lateral stiffness at crack/ ultimate load [kN/mm]	Failure types
TomoFix std. 1	4.1 / 5.4	0.6 / 1.2	3.1 / 5.0	1.8 / 2.9	1.3 /1.1	1 and 2
TomoFix std. 2	5.1 / 5.2	1.0 / 1.1	4.2 / 4.4	2.5 / 2.6	1.2 / 1.2	1 and 2
**Mean:**	**4.6 / 5.3**	**0.8 / 1.2**	**3.7 / 4.7**	**2.1 / 2.8**	**1.3 / 1.1**	
**SD ±:**	**0.7 / 0.1**	**0.3 / 0.1**	**0.8 / 0.4**	**0.5 / 0.2**	**0.1 / 0.1**	
PEEKPower 1	- / 3.7	- / 0.5	- / 2.9	- / 1.6	- / 1.3	1 and 2
PEEKPower 2	4.2 / 5.1	0.1 / 0.1	2.7 / 3.3	1.3 / 1.5	1.6 / 1.5	1 and 2
**Mean:**	**- / 4.4**	**- / 0.3**	**- / 3.1**	**- / 1.6**	**- / 1.4**	
**SD ±:**	**- / 0.1**	**- / 0.3**	**- / 0.3**	**- / 0.1**	**- / 0.1**	
iBalance 1	- / 5.7	- / 0.3	- / 1.6	- / 0.6	- / 3.6	2
iBalance 2	- / 5.4	- / 0.3	- / 2.1	- / 1.1	- / 2.6	2
**Mean:**	**- / 5.5**	**- / 0.3**	**- / 1.9**	**- / 0.9**	**- / 3.1**	
**SD ±:**	**- / 0.2**	**- / 0**	**- / 0.4**	**- / 0.4**	**- / 0.7**	
TomoFix sm 1	3.1 / 3.2	0.6 / 0.9	1.3 / 1.8	0.9 / 1.3	2.4 / 1.8	2
TomoFix sm 2	3.2 / 3.6	0.4 / 0.6	1.6 / 2.3	0.9 / 1.4	2.0 / 1.6	2
**Mean:**	**3.2 / 3.4**	**0.5 / 0.8**	**1.5 / 2.1**	**0.9 / 1.4**	**2.2 / 1.7**	
**SD ±:**	**0.1 / 0.3**	**0.1 / 0.2**	**0.2 / 0.4**	**0 / 0.1**	**0.3 / 0.1**	
Contour Lock 1	2.4 / 3.2	0.6 / 0.5	2.5 / 3.9	1.5 / 2.1	1.0 / 0.8	1 and 2
Contour Lock 2	- / 3.9	- / 0.5	- / 4.2	- / 2.2	- / 0.9	1 and 2
**Mean:**	**- / 3.6**	**- / 0.5**	**/ 4.1**	**- / 2.2**	**- / 0.9**	
**SD ±:**	**- / 0.5**	**- / 0**	**/ 0.2**	**- / 0.1**	**- / 0.1**	
Activmotion 1	- / 8.9	- / 1.3	- / 2.5	- / 0.6	- / 3.6	2
Activmotion 2	3.7 / 7.5	0.7 / 2.1	2.6 / 5.1	0.9 / 1.4	1.4 / 1.5	1 and 2
**Mean:**	**- / 8.2**	**- / 1.7**	**- / 3.8**	**- / 1.0**	**- / 2.6**	
**SD ±:**	**- / 0.7**	**- / 0.4**	**- / 1.3**	**- / 0.4**	**- / 1.1**	
FlexitSystem 1	- / 2.9	- / 1.0	- / 2.5	- / 1.7	- / 1.2	1 and 2
FlexitSystem 2	- / 4.5	- / 1.2	- / 2.7	- / 1.9	- / 1.7	1 and 2
**Mean:**	**- / 3.7**	**- / 1.1**	**- / 2.6**	**- / 1.8**	**- / 1.4**	
**SD ±:**	**- / 0.8**	**- / 0.1**	**- / 0.1**	**- / 0.1**	**- / 0.3**	

Displacements, valgus-malrotation of the tibia head and their corresponding crack and ultimate loads, including mean values and standard deviations (SD). The values of the first 6 groups were retrieved from our previous studies and reported here for purposes of comparison. The mean values and the standard deviation values are in bold

Overall, the highest average ultimate load, the point at which the specimens collapsed during the single loading to failure test, was 8.2 kN and occurred in group 6 (Activmotion). The Contour Lock 1 and 2 specimens showed the largest average lateral displacement (4.1 mm) at fracture of the lateral cortex. The iBalance group showed the highest lateral stiffness at ultimate load (3.1 kN/mm).

For all implant types, the average displacement on the medial side compared to the lateral side was always smaller. The determined valgus-malrotation of the tibia head was greater than, or equal to, the fixed limit of 1.4° of the permanent deflection angle for all implants with the exception of the iBalance and Activmotion specimens, which showed mean values 0.9 ° and 1° respectively. The TomoFix std. group showed the maximal valgus-malrotation at collapse time of the contralateral cortex (2.8 °).

The differences that were observed between the FlexitSystem group and the other groups, regarding the investigated parameters, were in majority statistically non-significant. The differences in the cases of valgus-malrotation and lateral displacement were statistically significant compared to the TomoFix std. and the Contour Lock, respectively (Table 6).

**Table 6:** *p*-Values obtained from the t-tests comparing the previously tested implants to the FlexitSystem

Groups	Ultimate load	Medial displ. at ultimate load [*mm*]	Lateral displ. at ultimate load [*mm*]	valgus-malrotation at ultimate load	Lateral stiffness at ultimate load
TomoFix std	> 0.05	> 0.05	> 0.05	< 0.05	> 0.05
PEEKPower	> 0.05	> 0.05	> 0.05	> 0.05	> 0.05
iBalance	> 0.05	> 0.05	> 0.05	> 0.05	> 0.05
TomoFix sm	> 0.05	> 0.05	> 0.05	> 0.05	> 0.05
Contour Lock	> 0.05	> 0.05	< 0.05	> 0.05	> 0.05
Activmotion	> 0.05	> 0.05	> 0.05	> 0.05	> 0.05

Mean values were compared. All statistical tests were performed two sided. Statistical significance was considered at *p* <  0.05.

### Fatigue loading to failure

**Fig. 5:**
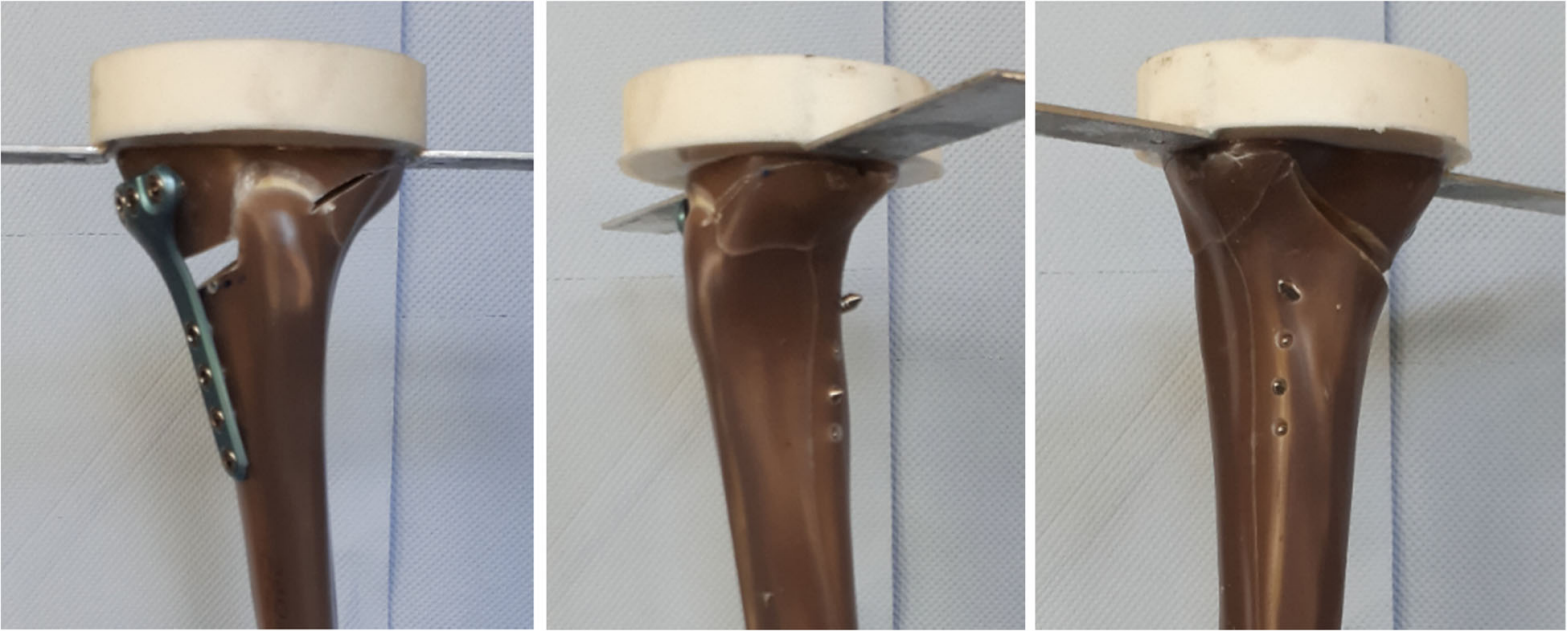
Fracture of the lateral cortex during cyclical testing (FlexitSystem 4). The opposite cortex appeared to be the weak point of the bone-implant constructs, as it was already observed during the static testing

The fracture of the FlexitSystem specimens subjected to cyclical tests occurred at the lateral cortex (Fig. 5), as with the static tests. If cracks occurred prior to the final failure of the specimens, they were generally not observable. The plates and screws remained undamaged during the cyclical testing. The tibia head of the specimens rotated counterclockwise, indicating a valgus-malrotation, which was also observed during the static tests.

A type 3 failure, which was checked by means of the maximal displacement range within hysteresis loops, did not occur in the FlexitSystem group, as well as in groups I, II,III and VI. This failure type occurred only in the TomoFix sm and Contour Lock groups (Diffo Kaze et al. 2017).

**Fig. 6:**
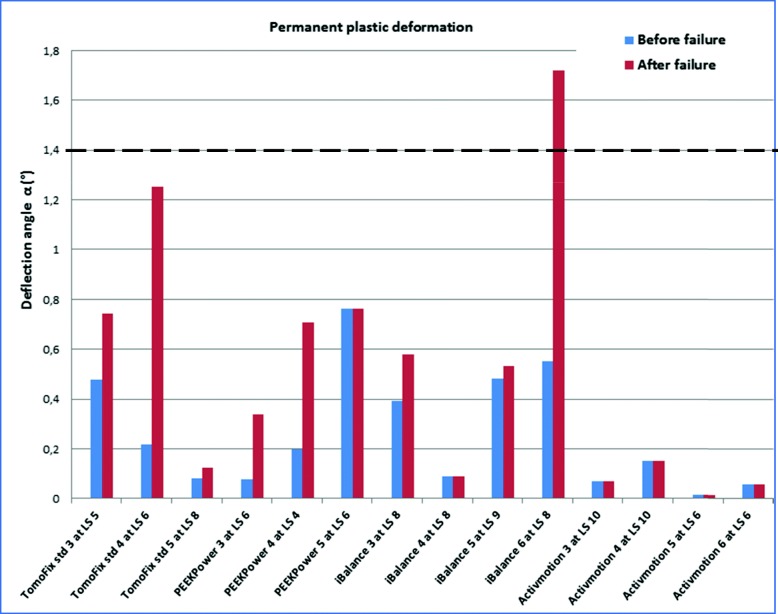
Deflection angle or valgus-malrotation of the tibia head before and after the failure for groups I, II, III and VI. The failure type 1 was observed in the case of the specimen iBalance 6 after the collapse of the opposite cortex. LS “n” means the failure occurred at load step “n”. The values of the first 3 groups were retrieved from our previous studies. Same values were considered before and after the failure for the Activmotion group

**Fig. 7:**
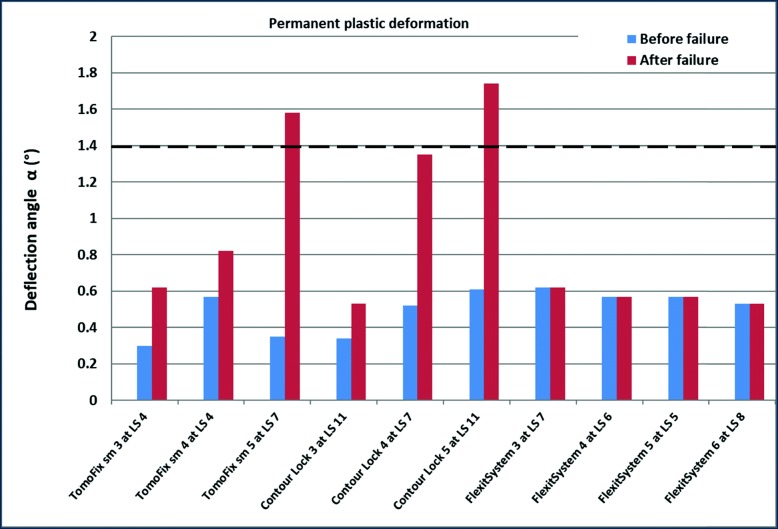
Deflection angle or valgus-malrotation of the tibia head before and after the failure for groups IV, V (From our previous studies) and VII. The same values were considered before and after the failure for group 7. A type 1 failure was observed for the specimens TomoFix sm 5 and Contour Lock 5

Figure 6 shows the values of the permanent plastic valgus-malrotation for groups I, II,III and VI from our previous study (Diffo Kaze et al. 2017). Figure 7 shows the permanent plastic deflection angle in groups IV, V and VI. The load history was indicated with the Load Step number (LSn) at which the failure occurred. A permanent plastic deflection angle after the failure was not determined in the FlexitSystem group, as the test was stopped immediately as the failure occurred. Therefore, the value after the failure was assumed to be equal to the value that was obtained before the failure. A type 1 failure, which was characterised by a permanent plastic deflection angle greater than 1.4 °, occurred only in the iBalance, TomoFix sm and Countour Lock groups, but after the failure of the specimen.

The deflection angles prior to the gross failure in the FlexitSystem group were, on average, higher than in the other groups.

For the sake of comparison, the results of the fatigue tests from our previous studies were summarised, together with the results that were obtained from the testing on the FlexitSystem plate (Table 7). This summary lists the maximal compressive force, the lateral and vertical stiffness of the specimens at the beginning of the first load step, the minimal number of cycles performed prior to the failure, and the types of failure. Average values and standard deviations in each group are given in Table 8.

**Table 7:** Summary of fatigue failure tests

Specimens	Maximal load [N]	Vertical stiffness K_V_ [N/mm]	Lateral stiffness K_L_ [N/mm]	Number of cycles	Failure types
TomoFix std. 3	1280	1350	2000	> 60,000	2
TomoFix std. 4	1440	2000	2500	> 80,000	2
TomoFix std. 5	1760	2500	2200	> 120,000	2
PEEKPower 3	1440	2000	2500	> 80,000	2
PEEKPower 4	1280	1950	2140	> 60,000	2
PEEKPower 5	1440	2785	2250	> 80,000	2
iBalance 3	1760	4000	3600	> 120,000	2,4
iBalance 4	1760	3000	3400	> 120,000	2
iBalance 5	1920	3000	2952	> 140,000	2
iBalance 6	1760	3500	2500	> 120,000	1,2
TomoFix sm 3	1280	2200	2000	> 60,000	2,3
TomoFix sm 4	1280	1750	1500	> 60,000	2,3
TomoFix sm 5	1760	2000	2300	> 120,000	1,2
Contour Lock 3	2400	2100	4400	> 200,000	2
Contour Lock 4	1760	2300	2400	> 120,000	2
Contour Lock 5	2400	2700	2600	> 200,000	1,2,3
Activmotion 3	2240	2500	6300	> 180,000	2
Activmotion 4	2240	2500	2900	> 180,000	2
Activmotion 5	1600	2500	4750	> 100,000	2
Activmotion 6	1600	3100	5100	> 100,000	2
FlexitSystem 3	1760	1070	2050	> 120,000	2
FlexitSystem 4	1600	960	1630	> 100,000	2
FlexitSystem 5	1440	950	1580	> 80,000	2
FlexitSystem 6	1920	960	1730	> 140,000	2

Maximal load, vertical & lateral stiffnesses, Min number of cycles (all values prior to failure) and failure types. The values of the groups I to VI were retrieved from our previous studies and reported here for the sake of comparison.

**Table 8:** Average mean values, including the standard deviations (SD), per group of the cyclic fatigue to failure tests

Groups	Maximal load [kN]		Vertical stiffness K_V_ [N/mm]		Lateral stiffness K_L_ [N/mm]		Number of cycles prior to failure	
	Mean	SD ±	Mean	SD ±	Mean	SD ±	Mean	SD ±
TomoFix std	1.5	0.2	1950	577	2233	252	> 86,000	30,550
PEEKPower	1.4	0.1	2245	468	2297	184	> 73,000	11,500
iBalance	1.8	0.1	3375	479	3113	490	> 125,000	10,000
TomoFix sm	1.4	0.3	1983	184	1933	330	> 80,000	28,300
Contour Lock	2.2	0.4	2367	250	3133	900	> 173,000	37,700
Activmotion	1.9	0.3	2650	260	4763	1219	> 140,000	40,000
FlexitSystem	1.7	0.2	985	49	1748	183	> 110,000	32,660

The values of the first 5 groups were retrieved from our previous studies and reported here for purposes of comparison (all comma values rounded to the 1st decimal).

For groups I, II, VI and VII only a type 2 failure was observed (Table 7). A damage of the fixation system occurred in the iBalance group. The FlexitSystem group showed the smallest stiffness values. The Contour Lock showed the highest average maximal load and number of cycles prior to failure.

Regarding the parameters investigated for the fatigue loading to failure tests, the Contour Lock group showed the highest values followed by the Activmotion group. The highest lateral and medial stiffness was observed in the Activmotion and iBalance groups respectively. The PEEKPower group showed higher stiffnesses than the TomoFix plates.

**Fig. 8:**
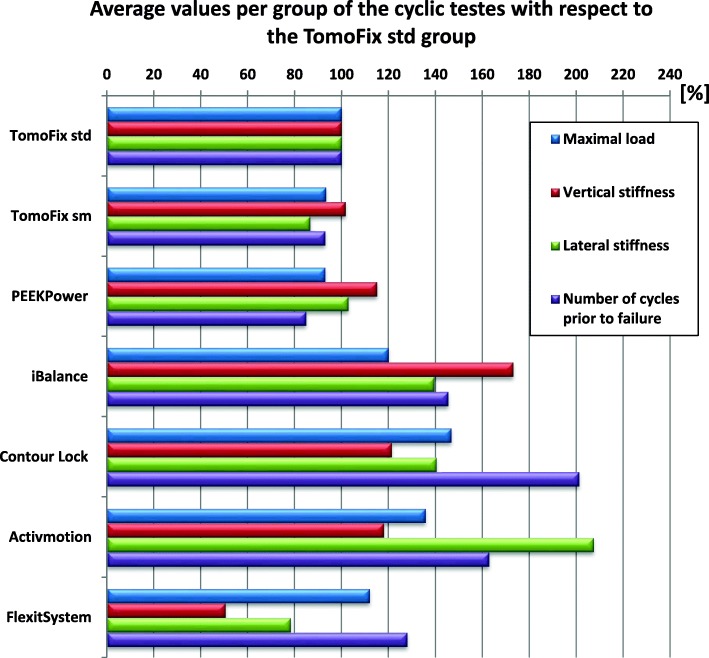
Average relative strength values of Table 6. The TomoFix std. group has been taken for reference

For better comparison with the TomoFix std., which is the gold standard plate, Fig. 8 shows the average relative values for the groups during the cyclic tests. These were calculated based on Table 8 and by taking the TomoFix std. group as a reference. The life span of the Contour Lock specimens prior to failure was, on average, twice as long as for the TomoFix std. specimens. The vertical stiffness of the iBalance group was, on average, around 1.7 time higher than that of the TomoFix std. group. The lateral stiffness of the Activmotion group was more than twice that of the TomoFix std. group. Regarding the lifespan during the test, the FlexitSystem plates were superior to the TomoFix and PEEKPower plates.

The differences that were observed between the FlexitSystem group and the other groups, regarding the ultimate load and number of cycles prior to failure, were statistically non-significant. The differences between the FlexitSystem group and the other groups, regarding vertical stiffness, were statistically significant, except for the comparison with the TomoFix std. group. Regarding lateral stiffness, the differences were statistically significant between the FlexitSystem group and each of the following groups: PEEKPower, iBalance and Activmotion (Table 9).

**Table 9:** *p*-Values obtained from the t-tests comparing the FlexitSystem to the previously tested implants

Groups	Ultimate load	Vertical stiffness [*mm*]	Lateral stiffness [*mm*]	Number of cycles prior to failure
TomoFix std	> 0.05	> 0.05	> 0.05	> 0.05
PEEKPower	> 0.05	< 0.05	< 0.05	> 0.05
iBalance	> 0.05	< 0.05	< 0.05	> 0.05
TomoFix sm	> 0.05	< 0.05	> 0.05	> 0.05
Contour Lock	> 0.05	< 0.05	> 0.05	> 0.05
Activmotion	> 0.05	< 0.05	< 0.05	> 0.05

Mean values were compared. Statistical significance was considered at *p* < 0.05.

## Discussion

The mechanical strength provided by the FlexitSystem plate for MOWHTO was investigated and compared to the mechanical strength of the other implants for MOWHTO, which were investigated in our previous studies. The same experimental setup and protocol was used in order to assess the mechanical strength of the implants. Hence, it was possible to compare the FlexitSystem plate to the previously tested implants, which were the following: The TomoFix std. plate, the PEEKPower plate, the iBalance implant, the Contour Lock HTO plate, the TomoFix sm plate and the Activmotion plate. (Maas et al. 2013; Diffo Kaze et al. 2015; Diffo Kaze 2016; Diffo Kaze et al. 2017). The loading of the specimens that was considered for the experimental setup corresponded to a realistic loading of the lower limb during the loading response phase of slow walking (Diffo Kaze et al. 2018). The key findings of the present study were the following: (1) The FlexitSystem plate showed sufficient strength for static loading, and average fatigue strength was comparable to the previously tested implants. (2) Full loading of the knee directly after osteotomy with the FlexitSystem plate and all the other tested implants should be avoided.

The FlexitSystem specimens failed due to the collapse of the opposite cortex, regardless of whether a static or cyclic failure test was applied, as was hypothesised. The same behaviour was observed in our previous studies (Maas et al. 2013; Diffo Kaze et al. 2015; Diffo Kaze 2016; Diffo Kaze et al. 2017) and other studies (Spahn and Wittig 2002; Stoffel et al. 2004; Agneskirchner et al. 2006; Watanabe et al. 2014). The displacements of the lateral side of the osteotomy were more pronounced than the medial displacement, which explains the valgus malrotation of the tibial head in the frontal plane during the static and cyclic loading tests. This behaviour may be related to the fact that the medial side was stiffer than the lateral side of the tibia head, as a result of the implant, which was fixed on the medial side.

During the static loading to failure test, the average ultimate force of the FlexitSystem specimens was 3.7 kN. This value was higher than the physiological vertical tibiofemoral contact force while slow walking, which is about 3 times the body weight (Taylor et al. 2004; Heinlein et al. 2009), e.g. 2.4 kN for a patient weighing 80 kg. This ultimate force obtained for the FlexitSystem group was comparable to the average values of the Contour Lock and TomoFix sm groups. However, it was smaller compared to the average values of the PEEKPower, TomoFix std., iBalance and Activmotion groups (4.4 kN, 5.3 kN, 5.5 kN and 8.2 kN, respectively).

It is important to use implants that will avoid fracture of the cortical lateral hinge prior to the beginning of gap healing, as patients with a fracture of the lateral cortex after MOWHTO exhibit delayed union (Schröter et al. 2015; Takeuchi et al. 2012). Gap healing starts approximately after 3 to 8 weeks (Marsell and Einhorn 2011). Considering that a healthy active person performs 1 million loading cycles of their limb per year (Baleani et al. 2003; Bergmann et al. 2001; Thielen 2009), 3 weeks corresponds approximately to 60,000 loading cycles. This means that the FlexitSystem and all the previously tested implants, would preserve a safe lateral cortex for at least 3 weeks, as the lowest number of cycles was 73,000 which was the mean number of cycles for the PEEKPower group (Table 8). However, the maximal load at failure that was observed during the fatigue tests for the FlexitSystem group was, on average, 1.7 kN, which is smaller than the threshold value of 2.4 kN for physiological loading. This average maximal load at failure obtained for the FlexitSystem group was higher than those of the TomoFix and PEEKPower groups, and smaller than those of the iBalance, Activmotion and Contour Lock groups. The Contour Lock showed the highest maximal load at failure of 2.2 kN on average, which was smaller than the threshold value of 2.4 kN.

A valgus deformation of the knee will result from the valgus malrotation of the tibial head, something which did occur during the tests. Consequently, the localisation of the mechanical axis and the primarily performed correction will be altered. No permanent plastic valgus-malrotation of the tibia head, which led to a type 1 failure before collapse of the contralateral cortex, was observed in all the tested group. Permanent plastic valgus malrotations resulting in a type 1 failure after fracture of the contralateral cortex were observed in the iBalance, TomoFix sm and Contour Lock groups, as shown in Figs. 6 and 7. Although the deflection angles prior to collapse of the specimens were small, they were the highest, on average, in the FlexitSystem group. This suggests that all other implants conserve the correction angle prior to collapse of the specimen better than the FlexitSystem plate. It is cautioned at this level that the last observation is only valid if there is no bone healing prior to the fatigue failure, which is not a realistic scenario. The values of the permanent plastic valgus malrotation after the final fracture of the contralateral cortex were not determined, but just assumed, in the FlexitSystem group (Fig. 7). This is because the testing was stopped directly after the collapse of the specimens in order to avoid damage of the displacement sensors.

No type 3 failures were observed for the FlexitSystem specimens. This suggests that the FlexitSystem plate offered good stability to the bone-implant construct as a type 3 failure quantifies the wobble degree of the bone-implant construct. Compared to our previous studies, only the TomoFix sm and Contour Lock groups showed type 3 failures, suggesting the superiority of the FlexitSystem compared to the TomoFix sm and the Contour Lock, regarding this parameter. However, this failure type occurred in the Contour Lock group after higher loading cycles than in the FlexitSystem group (Maas et al. 2013).

Stiffness was investigated as an additional damage indicator,with high stiffness of the lateral side of the bone-implant construct suggesting a stable lateral cortical hinge. The FlexitSystem group showed the minimal lateral stiffness, 1748 N/mm on average, compared to the other groups. On the other hand, the Activmotion group showed the maximal lateral stiffness, 4763 N/mm on average. These observations highlight the influence of the plate positioning on the stability of the lateral cortical hinge, as the Activmotion plate is positioned onto the antero-medial side of the tibia head, whereas the FlexitSystem and the other previously tested plates are centred on the medial side of the tibia head.

We concluded in our previous studies (Maas et al. 2013; Diffo Kaze et al. 2015) that mechanical static and fatigue strength increases with a wider proximal T-shaped plate design together with diverging proximal screws, as used in the Contour Lock plate, or in a closed-wedge construction as with the iBalance design. This conclusion was confirmed by the results of the FlexitSytem, thus also confirming our hypothesis. However, since mechanical stimulation can induce fracture healing or alter its biological pathway (Claes et al. 1997; Claes et al. 1998; Goodship and Kenwright 1985; Isaksson 2012), the clinical performance of implants should not be only correlated to their mechanical performance in terms of high mechanical strength. It is a necessary condition to have a minimum stability for the functionality of the bone implant-constructs.

Diffo Kaze et al. simulated the following different loading conditions of the lower limb after HTO: (1) realistic loading conditions, including muscle forces, during slow walking and (2) simplified loading conditions consisting of a vertical loading of the tibia plateau, like for the experimental testing. The study showed that the stress distributions in the HTO-implants that were obtained in loading conditions (1) and (2) were comparable to one another. The stresses in the implants were all lower than the threshold stress values of each implant material (Diffo Kaze et al. 2018). This meant that loading the bone-implant constructs with physiological loads would not lead to critical stresses that could cause implant damage. This observation is, however, only valid if the technical recommendations of the implant fixation are respected during the HTO procedure.

Limitations of this study are the limited number of specimens per group and the fact that bone healing normally takes place a few days postoperatively, before high loading cycle numbers are reached. Hence, one should proceed cautiously when transferring the present results to clinical settings.

## Conclusion

The FlexitSystem plate showed sufficient strength for static loading and average fatigue strength compared to the previously tested implants. For higher flexibility of the bone-implant constructs after surgery, the FlexitSystem plates should be chosen. Full body dynamic loading of the tibia after MOWHTO with the investigated implants should be avoided for at least 3 weeks. Implants with a wider T-shaped proximal end, positioned onto the antero-medial side of the tibia head, or inserted in the osteotomy opening in a closed-wedge construction, provided higher mechanical strength than implants with a small T-shaped proximal end, centred onto the medial side of the tibia head.

## Abbreviations

LSn: Load step number
MOWHTO: medial open-wedge High Tibial Osteotomy
PEEK: Polyetheretherketon
SD: Standard deviation
sm: Small stature
std: Standard

## References

[CR1] Agneskirchner J, Freiling D, Hurschler C, Lobenhoffer P (2006). Primary stability of four different implants for opening wedge high tibial osteotomy. Knee Surg Sports Traumatol Arthrosc.

[CR2] Amendola A, Bonasia D (2010). Result of high tibial osteotomy: review of literature. Int Orthop.

[CR3] Baleani M, Traina F, Toni A (2003). The mechanical behaviour of a pre-formed hip spacer. Hip Int.

[CR4] Bergmann G, Deuretzbacher G, Heller M, Graichen F, Rohlmann A, Strauss J, Duda G (2001). Hip contact forces and gait patterns from routine activities. J Biomech.

[CR5] Brinkman J-M, Lobenhoffer P, Agneskirchner J, Staubli A, Wymenga A, van Heerwaarden R (2008). Osteotomies around the knee: patient selection, stability of fixation and bone healing in high tibial osteotomy. J Bone Joint Surg Br.

[CR6] Claes L, Augat P, Suger G, Wilke H (1997). Influence of size and stability of the osteotomy gap on the success of fracture healing. J Orthop Res.

[CR7] Claes L, Heigele C, Neidlinger-Wilke C, Kaspar D, Seidl W, Margevicius K, Augat P (1998) Effects of mechanical factors on the fracture healing process. Clin Orthop Relat Res 355;132–147 10.1097/00003086-199810001-00015 9917634

[CR8] Diffo Kaze A (2016). Etude biomécanique comparative de cinq différents systèmes de fixation utilisés dans les cas d'ostéotomies tibiales valgisantes: Essais expérimentaux et simulations numériques incluant les forces musculaires.

[CR9] Diffo Kaze A, Maas S, Belsey J, Hoffmann A, Pape D (2017) Static and fatigue strength of a novel anatomically contoured implant compared to five current open-wedge high tibial osteotomy plates. J Orthop 4(39). 10.1186/s40634-017-0115-3 10.1186/s40634-017-0115-3 PMC5722784 29222607

[CR10] Diffo Kaze A, Maas S, Kedziora S, Belsey J, Haupert A, Hoffmann A, Pape D (2018) Numerical comparative study of five currently used implants for high tibial osteotomy: realistic loading including muscle forces versus simplified experimental loading. J Exp Orthop 5(28). 10.1186/s40634-018-0144-6 10.1186/s40634-018-0144-6 PMC6082749 30091026

[CR11] Diffo Kaze A, Maas S, Waldmann D, Zilian A, Dueck K, Pape D (2015) Biomechanical properties of five different currently used implants for open-wedge high tibial osteotomy. J Exp Orthop 2(14). 10.1186/s40634-015-0030-4 10.1186/s40634-015-0030-4 PMC4538734 26914882

[CR12] Goodship A, Kenwright J (1985). The influence of induced micromovement upon the healing of experimental tibial fractures. J Bone Joint Surg Br.

[CR13] Heinlein B, Kutzner I, Graichen F, Bender A, Rohlmann A, Halder A, Beier A, Bergmann G (2009). ESB clinical biomechanics award 2008: complete data of total knee replacement loading for level walking and stair climbing measured in vivo with a follow-up of 6-10 months. Clin Biomech.

[CR14] Isaksson H (2012). Recent advances in mechanobiological modeling of bone regeneration. Mech Res Commun.

[CR15] Lobenhoffer P, Agneskirchner J (2003). Improvements in surgical technique of valgus high tibial osteotomy. Knee Surg Sports Traumatol Arthrosc.

[CR16] Maas Stefan, Diffo Kaze Arnaud, Dueck Klaus, Pape Dietrich (2013). Static and Dynamic Differences in Fixation Stability between a Spacer Plate and a Small Stature Plate Fixator Used for High Tibial Osteotomies: A Biomechanical Bone Composite Study. ISRN Orthopedics.

[CR17] Marsell R, Einhorn T (2011). The biology of fracture healing. Injury.

[CR18] Pape D, Lorbach O, Schmitz C, Busch L, Van Giffen N, Seil R, Kohn DM (2010). Effect of a biplanar osteotomy on primary stability following high tibial osteotomy: a biomechanical cadaver study. Knee Surg Sports Traumatol Arthrosc.

[CR19] Pape D, Seil R, Adam F, Kohn D, Lobenhoffer P (2004). Bildgebung und präoperative Planung der Tibiakopfosteotomie. Orthopäde.

[CR20] Schröter S, Freude T, Kopp M, Konstantinidis L, Döbele S, Stöckle U, van Heerwaarden R (2015). Smoking and unstable hinge fractures cause delayed gap filling irrespective of early weight bearing after open wedge osteotomy. Arthroscopy.

[CR21] Spahn G, Kirschbaum S, Kahl E (2006). Factors that influence high tibial osteotomy results in patients with medial gonarthritis: a score to predict the results. Osteoarthr Cartil.

[CR22] Spahn G, Mückley T, Kahl E, Klinger H, Steinhauser E, Hofmann G (2007). Biomechanical investigation of uniplanar and biplanar cuts in opening-wedge high tibial osteotomy. BIOmaterialien.

[CR23] Spahn G, Wittig R (2002). Primary stability of various implants in tibial opening wedge osteotomy: a biomechanical study. J Orthop Sci.

[CR24] Stoffel K, Stachowiak G, Markus K (2004). Open wedge high tibial osteotomy: biomecanical investigation of the modified Arthrex osteotomy plate (Puddu plate) and the TomoFix plate. Clin Biomech (Bristol, Avon).

[CR25] Takeuchi R, Ishikawa H, Kumagai K, Yamaguchi Y, Chiba N, Akamatsu Y, Saito T (2012). Fractures around the lateral cortical hinge after a medial opening-wedge high tibial osteotomy: a new classification of lateral hinge fracture. Arthroscopy.

[CR26] Taylor W, Heller M, Bergmann G, Duda G (2004). Tibio-femoral loading during human gait and stair climbing. J Orthop Res.

[CR27] Thielen T (2009). Optimierung der Tragfähigkeit von antibiotikabeladenen PMMA Hüftinterimsprothesen.

[CR28] Watanabe K, Kamiya T, Suzuki D, Otsubo H, Teramoto A, Suzuki T, Yamashita T (2014). Biomechanical stability of open-wedge high tibial osteotomy: comparison of two locking plates. Open J Orthop.

